# Present and Future: Using Ecological Niche Modeling to Understand the Conservation Status of *Alouatta caraya* (Primates, Atelidae) and Promote Its Protection

**DOI:** 10.1002/ajp.70066

**Published:** 2025-08-19

**Authors:** Jéssyca B. Schwantes, Lucas A. Antunes, Vanessa B. Fortes, Lizandra J. Robe

**Affiliations:** ^1^ Programa de Pós‐Graduação em Biodiversidade Animal Universidade Federal de Santa Maria Santa Maria Rio Grande do Sul Brazil; ^2^ Laboratório de Primatologia Universidade Federal de Santa Maria Palmeira das Missões Rio Grande do Sul Brazil

**Keywords:** black‐and‐gold howler, climate change, fragmentation, habitat suitability, potential distribution

## Abstract

Climate change is one of the main drivers of shifts in species distributions. Ecological niche models (ENMs) are valuable tools for assessing these effects and informing conservation efforts. This study employed ENMs to assess the impact of climate change on the present (from 1970 to 2000) and future (up to 2100) climate suitability patterns of the black‐and‐gold howler monkey (*Alouatta caraya* [*A. caraya*]), which is facing serious threats due to habitat changes and disease, especially in the southernmost part of its range. We also evaluated the effectiveness of current protected sites for the species' conservation in the future. For each 20‐year interval, we used seven different algorithms and reconstructed a consensus map using ensemble techniques. We then reevaluated the geographical patterns of habitat suitability, accounting for dispersal restrictions and fragmentation history. Our results suggest that areas of high habitat suitability for *A. caraya* may be much smaller than the geographic distribution reported by the IUCN, with future projections predicting a continuous decrease in suitable areas from 2021 to 2100. Furthermore, most sites with high suitability for *A. caraya* are located outside protected areas, with < 11% of its potential distribution range currently under protection. The extent of protected areas further drops by nearly 50% when only areas that remain suitable for *A. caraya* over the next 80 years (refuges) are considered. Moreover, areas with higher suitability indices are clustered within the Chaco and Pampa regions, which have been subjected to significant habitat conversion during the last 35 years. Therefore, climate change and habitat conversion pose a significant threat to *A. caraya*'s effective conservation, warranting a review of its conservation status.

## Summary

- The high suitability area predicted for *A. caraya* in the present is much smaller than the geographic distribution range reported for the species by IUCN.
- Future projections suggest a continuous decrease in the high suitability area of the species until 2100. Stable regions able to maintain *A. caraya* populations over time present a low coverage of protected areas.
- Higher suitability areas for *A. caraya* are concentrated in regions such as Chaco and Pampa, subjected to significant habitat conversion.

## Graphical abstract

Free‐living *Alouatta caraya*: the male (top left) and his females (bottom left). We used ecological niche modeling to assess the species' climate suitability both now and in future projections. Between now and 2030, there is a 31% reduction in distribution. Environmental protection areas cover < 15% of the climatically suitable regions for the species. Stable regions capable of sustaining *A. caraya* populations over time have low protected area coverage.

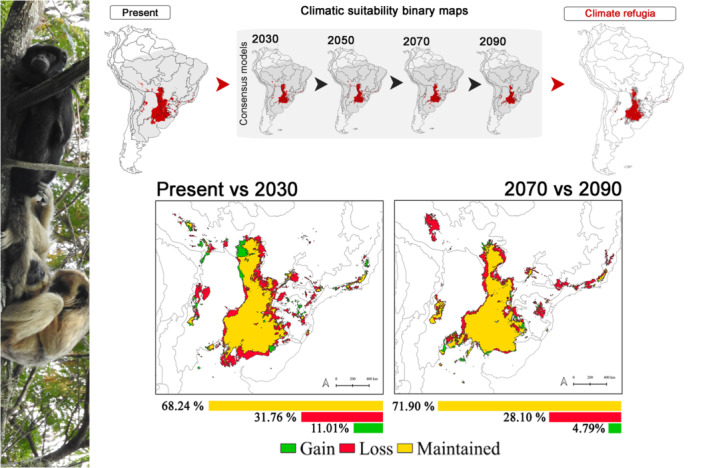

**Abbreviations**
AUC: area under the curve
BCC‐CSM2‐MR: Beijing Climate Center Climate System Model, Version 2, Medium Resolution
BRT: boosted regression tree
CI: connectivity index
CMCC‐ESM2: Centro Euro‐Mediterraneo Sui Cambiamenti Climatici Earth System Model Version 2
CMIP: coupled model intercomparison project
ENMs: ecological niche models
FI: fragmentation index
GAM: generalized additive model
GCM: global climate models
GLM: generalized linear model
HR: home range
IUCN: International Union for Conservation of Nature
MaxEnt: maximum entropy
Max(se + sp): maximizing sensitivity and specificity
MDD: maximum dispersal distance
PA: protected areas
RF: random forest
sd: standard deviation
SSP: shared socioeconomic pathways
SVM): support vector machine
TSS: true skill statistics
VIF: variance inflation factor
WDPA: World Database on Protected Areas
%PA: % of PA

## 1 Introduction

Climate change poses a significant global threat to biodiversity, as it can drastically alter habitats, disrupt species interactions, and drive species extinctions (Zhang et al. 2020). These changes may also alter the geographic distribution of species, causing either expansions or contractions in geographical ranges (Braz et al. 2019; Ebrahimi et al. 2021). A multitaxon meta‐analysis conducted on over 100 species predicted that 1 out of every 6 species would face the threat of extinction due to climate change (Urban 2015). Mammals are especially vulnerable to such changes, which can drastically reduce the distribution of several species, causing decreases and shifts in species‐rich areas (Ureta et al. 2022). This is especially worrisome because more than 1300 mammalian species are currently under threat of extinction, of which 348 are primates (IUCN 2022).

According to Fernández et al. (2022), over 65% of primate species fall into 1 of the top 3 International Union for Conservation of Nature (IUCN) Red List categories (Vulnerable, Endangered, and Critically Endangered) due to a combination of climate change and other human‐induced pressures. Currently, changes such as an increase in annual temperature are already adversely impacting several primate species, resulting in reduced range sizes and behavioral changes, such as modified resting patterns (Korstjens et al. 2010; Zhao et al. 2017; Zhang et al. 2020). Future impacts may become even worse, depending on factors such as dispersal abilities, resource availability, thermal regulation, physiological tolerance, and vulnerability to emerging pathogens (Schloss et al. 2012; Ameca y Juárez et al. 2012; Pacifici et al. 2015; Stewart et al. 2020; Carlson et al. 2022), which can vary across taxa. In the genus *Alouatta*, the mantled howler monkey (*Alouatta palliata*) and the Yucatan black howler monkey (*Alouatta pigra*) already ranked in the top 10 percent of species at greatest risk due to drastic reductions in suitable climatic and land cover conditions, demonstrating the high vulnerability of the genus to such changes (Ureta et al. 2022).

Ecological niche models (ENMs) provide valuable tools for exploring the impacts of climate change on species distributions. By correlating presence, presence–absence, or presence–background data with abiotic and biotic variables, these models can predict potential areas where a species could survive (Meyer et al. 2014; Cerasoli et al. 2022). ENMs can also be used to project habitat suitability for a species in past or future scenarios, offering insights into how its distribution may shift under varying conditions. Furthermore, they can help elucidate the environmental conditions necessary for population persistence, thus increasing the effectiveness of conservation measures (LaRue and Nielsen 2008; Zarate et al. 2023; Nogués‐Bravo 2009).

Previous research employing ENMs on the genus *Alouatta* has focused on analyzing a species' potential versus actual distribution, predicting or identifying potential areas of sympatry between species, and modeling priority actions for species conservation (Ortiz‐Martínez et al. 2008; Vidal‐García and Serio‐Silva 2011; Holzmann et al. 2015; Freire Filho and Palmeirim 2020; Povill et al. 2023; Vásquez‐Aguilar et al. 2024). Holzmann et al. (2015) employed ENMs to predict a wide potential distribution for the black‐and‐gold howler monkey (*Alouatta caraya* [*A. caraya*]), identifying a small area of sympatry between this species and the red howler monkey (*Alouatta guariba* [*A. guariba*]). Nevertheless, Oklander et al. (2017) demonstrated that human activities related to deforestation have isolated several populations of the former species. As a result, although the IUCN (2022) currently considers *A. caraya* a Near Threatened species, there are claims that its global classification should be upgraded to Vulnerable (Oklander et al. 2017). This is based on this species' high susceptibility to the yellow fever virus, coupled with an underestimation of its risk levels throughout its range. In the southernmost areas of its distribution, for example, experts have classified this species as Vulnerable (Argentina: Ojeda et al. 2012; Paraná state, Brazil: Decree No. 7,264/2010), Endangered (Rio Grande do Sul state, Brazil: Decree No. 51,797/2014), and even Critically Endangered (Santa Catarina state, Brazil: Santa Catarina State Environmental Council (CONSEMA), Resolution 02/2011) according to IUCN criteria.

To predict risks and mitigate their effects on the conservation of *A. caraya*, it is crucial to assess the species' environmental requirements and potential distribution areas under present and future scenarios. Accordingly, in this study, we aim to reconstruct habitat suitability patterns for this species over a broad timescale, projecting its potential response to climatic changes from the present (1970–2000) until 2100 in 20‐year intervals under different scenarios. We evaluated these patterns in the context of the dispersal constraints faced by the target species, as well as the historical fragmentation and isolation of its populations in some of their most climatically stable areas. Additionally, we compared these patterns with the current distribution of protected areas to evaluate their effectiveness in the future conservation of the species and to assess the effect of further habitat loss on the persistence of its populations.

## 2 Methods

We state that no ethical approval was required for this study, as it relied exclusively on previously obtained data and did not involve any handling or collection of live animals.

### 2.1 Occurrence Data

*A. caraya* is the most widespread species within its genus (Cortés‐Ortiz et al. 2015). It typically occupies a variety of habitats, including riparian zones, flooded areas, and semideciduous forests, and is found in both continuous and fragmented forests across South America, particularly in Brazil, Paraguay, Argentina, Bolivia, and Uruguay (Bicca‐Marques 1990; Oklander et al. 2010; Holzmann et al. 2015; Jardim et al. 2019; Gorostiaga et al. 2021). Throughout this region, we compiled occurrence points of *A. caraya* from various databases: Portal da Biodiversidade (https://portaldabiodiversidade.icmbio.gov.br/portal/), SALVE (CPB/ICMBio/Sistema de Avaliação do Estado de Conservação da Biodiversidade, 2022), Global Biodiversity Information Facility (GBIF) (https://www.gbif.org/), and SpeciesLink (http://splink.cria.org.br/). We conducted data cleaning in two steps. In the first step, we removed points located outside the recognized distribution range of the target species using QGIS 3.20.1‐Odense (QGIS Development Team 2021), based on the species' geographical distribution reported by the IUCN portal (https://www.iucnredlist.org/), with a buffer of 100 km. In the second step, we retained only one occurrence per grid cell, using the *cellFromXY* function from the Raster package (Hijmans 2023) in R version 4.2.2 (R Core Team 2023).

### 2.2 Bioclimatic Variables

We downloaded bioclimatic layers from WorldClim 2.1 database for 1970–2000 (hereafter referred to as “present”) and for different future periods, in 20‐year intervals (2021–2040 (hereafter 2030), 2041–2060 (hereafter 2050), 2061–2080 (hereafter 2070), and 2081–2100 (hereafter 2090) at a resolution of 2.5 arc‐min (i.e., ~4.5 km)). We used CMIP 6 data (Coupled Model Intercomparison Project) to obtain future projection models, selecting the two global climate models (GCM) that demonstrated the most consistent temperature increase patterns according to Hausfather et al. (2022): BCC‐CSM2‐MR and CMCC‐ESM2. For each of these, we employed three Shared Socioeconomic Pathways (SSP): 2–4.5 (optimistic), 3–7.0 (intermediate), and 5–8.5 (pessimistic). All six models followed stable temperature increase patterns ranging from 2°C to 5°C (Hausfather et al. 2022).

To avoid multicollinearity, from the set of 19 bioclimatic layers available in WorldClim 2.1, we selected variables presenting a variance inflation factor (VIF) lower than 9 (Quinn and Keough 2002, https://github.com/oliveirab/R-codes/blob/master/vif_func.R) and pairwise Pearson's correlation values lower than |0.6|. In both cases, we employed environmental data extracted for 10,000 background points, obtained in a radius of 1233 km (see below) from all trimmed records of *A. caraya*. As a result, we retained seven bioclimatic layers: Mean Diurnal Range (Mean of Monthly (Max–Min Temperature)) (bio 2), Isothermality (bio 3), Mean Temperature of Wettest Quarter (bio 8), Precipitation of Driest Month (bio 14), Precipitation Seasonality (Coefficient of Variation) (bio 15), Precipitation of Warmest Quarter (bio 18), and Precipitation of Coldest Quarter (bio 19).

### 2.3 Ecological Niche Models

For ecological niche modeling, we employed seven different algorithms, as available in the SDM package (Naimi and Araújo 2016) in R. These are classified into four categories: (1) presence‐only (Distance): BIOCLIM (Nix 1986); (2) presence‐background (Machine Learning): Maximum Entropy (MaxEnt) (Phillips et al. 2006); (3) presence–absence (Machine Learning): Boosted Regression Tree (BRT) (Hijmans et al. 2017), Random Forest (Mi et al. 2017), and Support Vector Machine (SVM) (Betancourt 2005); (4) presence–absence (Regression): Generalized Linear Model (GLM) and Generalized Additive Model (GAM) (Guisan et al. 2002). For each model, we randomly generated 10 bootstrap replicates, using 70% of the trimmed occurrences for training and 30% for testing. Finally, we projected the suitability patterns obtained for the present onto different future models, scenarios, and time periods.

We estimated the background area using a buffer of 1233 km around the set of records, based on the mean distance between the most peripheral points and the centroid of the convex hull created for the target species using the Alpha Shapes Tool in QGIS 3.20.1‐Odense software. The number of background points was set to 10 times the number of occurrence points. Additionally, as different studies suggest that *Alouatta* species are generally parapatric (Cortés‐Ortiz et al. 2003; Schwantes et al. 2025), we employed occurrence points of other *Alouatta* species as absence records for the target species. For this task, we downloaded records of *Alouatta belzebul*, *A. guariba*, *Alouatta sara*, *Alouatta seniculus*, and *Alouatta ululata* from the same databases described above and generated a matrix of absence records. This matrix was further trimmed to remove points within a radius of 100 km from the recognized distribution of *A. caraya*, to avoid including potentially overlapping areas or records related to misidentifications.

We evaluated the performance of each model using the area under the curve (AUC) and the true skill statistics (TSS), with AUC > 0.8 and TSS > 0.5 indicating excellent and good predictive power, respectively (Pearce and Ferrier 2000; Allouche et al. 2006). We calculated the mean relative importance of each variable in the modeling strategies using the *getVarImp* function, under two approaches: the Pearson correlation and the AUC metric. After evaluating the models, we estimated a consensus using the *ensemble* function, based on the median suitability values recovered by each algorithm. Subsequently, we transformed suitability maps into binary maps based on the maximum sensitivity + specificity threshold value, as calculated from the option max(se + sp). All these analyses were conducted in R's SDM package.

To deal with model uncertainties and increase the consistency of the results, we summarized future reconstructions obtained by each of the six models for each 20‐year interval using the *ensemble* function with the same parameters described above. For this task, we first reconstructed consensus models between different GCM's and then between different SSP projections. Afterwards, we summarized the final binary models for each future 20‐year interval using a threshold of 0.34, in which an area was deemed potentially occupied in the future only if it was identified as suitable by at least two out of the three primary SSP projection models (Figure S1).

### 2.4 Maximum Dispersal Extent

We estimated the potential dispersal constraints of the target species following the procedures described by Bowman et al. (2002), measuring the maximum dispersal distance (MDD) and then establishing buffers for the current or predicted ranges. For this task, we first estimated the MDD in one generation using Equation (1), with a home range (HR) of 11.875 ha, as obtained for *A. caraya* by Galán‐Acedo et al. (2019).

(1) $$\mathrm{MDD}\;\mathrm{in}\;\mathrm{one}\;\mathrm{generation}=40\cdot \sqrt{H}R\cdot 10,000.$$

To measure the MDD in fixed intervals, we employed a generation time of ±10 years (Pacifici et al. 2013). Thus, for example, from 2020 to 2040, there were 20 years and 2 generations; from 2020 to 2100, there were 80 years, and 8 generations. We then used these estimates to calculate the MDD for each of these intervals using Equation (2):

(2) MDD=MDDinonegeneration*numberofgenerations

### 2.5 Landscape Metrics

In areas with the highest habitat suitability values, we obtained data on land use and habitat conversion from the MapBiomas project (http://brasil.mapbiomas.org). In this context, we evaluated the period from 1985 to 2020 in 7‐year intervals, considering the rates of deforestation and land clearing in the American Gran Chaco, South American Pampa, and Brazil.

We also calculated the fragmentation and connectivity indices (FI and CI, respectively) within the most suitable areas (Chacoan and Pampean provinces of the Chacoan Dominion, as defined by Morrone et al. (2022)) using layers obtained from the MapBiomas project. For this task, we considered only areas including woody natural vegetation types since howler monkeys depend on trees and shrubs for movement, feeding, and resting/sleeping, and do not inhabit purely herbaceous environments.

First, we employed the models accounting for MDD in 7‐year intervals to clip the MapBiomas rasters (see Section 2.4). After clipping, we converted rasters to vectors using the *polygonize* tool. We then calculated each woody natural fragment's area and perimeter values using the QGIS field calculator tool. Fragments smaller than 11.875 ha were excluded, following the minimum HR for the species proposed by Galán‐Acedo et al. (2019).

We calculated FI according to Equation (3), as the ratio between the total woody area and the total woody perimeter. In this case, decreasing values of FI suggest increasing fragmentation.

(3) $$\text{FI}=\frac{\Sigma \;WoodyAREAyear}{\Sigma \;WoodyPERIMETERyear}$$

To infer CI, we measured the mean distance between the centroids of each pair of woody fragments using the *pointDistance* function of the Raster package in R. After calculating the average distance values for each fragment, we combined these values into an overall mean for each evaluated year. Thus, for CI, increasing values indicate decreasing connectivity.

### 2.6 Area Calculations

We employed the *intersection* and *difference* tools available in QGIS 3.20 to calculate how much of the occupied or potentially occupied area was maintained, gained, and lost during different periods. We obtained these measures by comparing each future scenario with the present, as well as each pair of successive future scenarios. In all cases, we employed the consensus binary models generated in the previous steps (see more in Session 2.3).

Additionally, we estimated the percentage of the potentially suitable areas recovered by our binary models that include protected areas (PA) for each of the countries where the species has been recorded (namely, Argentina, Brazil, Uruguay, Paraguay, and Bolivia) for the present (1970–2000), and each future 20‐year interval (2030, 2050, 2070, and 2090). Such values were also obtained for the clipped “stable areas,” defined as regions of the geographical space that sustain a potential presence in our binary models across different periods. Furthermore, we also measured the percentage of PA (%PA) considering the MDD to estimate the potential long‐term safeguarding of habitats if the species fully utilizes its dispersal potential. We obtained the shapefiles of the protected areas from the World Database on Protected Areas platform (WDPA, https://www.protectedplanet.net/en) and performed all data cleaning and calculations in QGIS 3.20.1‐Odense.

## 3 Results

### 3.1 Models and Variables

Initially, we compiled 1814 records for *A. caraya* from different databases. From these, we retained 710 after the 2 trimming steps. All ENMs achieved AUC and TSS values greater than 0.79 and 0.47, respectively (Table 1). The analysis of variable importance showed that bio 3 (=Isothermality; 52.1%), bio 2 (=Mean Diurnal Range; 29.9%), and bio 14 (=Precipitation of Driest Month; 25.1%) were the most important variables for the models (Figure S2A).

**Table 1:** Mean performance of the seven modeling algorithms, as estimated by the area under the curve (AUC) and the true skill statistics (TSS), based on 10 bootstrap replicates using 30% of the trimmed records as test data.

Algorithm	AUC (sd)	TSS (sd)	Max(se + sp)
Bioclim	0.81 (0.011)	0.47 (0.020)	0.047
BRT	0.86 (0.012)	0.57 (0.018)	0.077
GAM	0.88 (0.008)	0.61 (0.021)	0.123
GLM	0.79 (0.008)	0.49 (0.016)	0.126
MaxEnt	0.89 (0.008)	0.62 (0.021)	0.415
SVM	0.89 (0.013)	0.62 (0.029)	0.085
Random Forest	0.97 (0.005)	0.84 (0.021)	0.136

*Note:* Maps were binarized using the maximum sensitivity and specificity (Max(se + sp)) threshold.

Abbreviations: sd = standard deviation, BRT = Boosted Regression Tree, GAM = Generalized Additive Model, GLM = Generalized Linear Model, MaxEnt = Maximum Entropy, SVM = Support Vector Machine.

According to the response curves obtained for the bioclimatic layers, bio 3 (=Isothermality), bio 14 (=Precipitation of Driest Month), and bio 19 (=Precipitation of the Coldest Quarter) showed the greatest variation in habitat suitability values (Figure S2B). Nevertheless, they presented opposing response patterns, with increasing suitability values toward higher values for bio 14 (with a peak between 150 and 250 mm), but toward lower values for bio 3 (with a peak near 50) and bio 19 (peak near 0 mm). Bio 2 (=Mean Diurnal Range) presented a clear unimodal curve toward intermediate values.

Regarding future projections, the BCC‐CSM2‐MR models generally showed higher maintenance areas when compared to the CMCC‐ESM2 models. Consequently, with the first GCM, different periods showed a lower percentage of area loss and a higher percentage of area gain. Except for the first 20 years, within each GCM, the SSP 2‐4.5 projections showed the highest mean maintenance area (Table S1).

### 3.2 Area Calculations and Habitat Suitability

**Figure 1:**
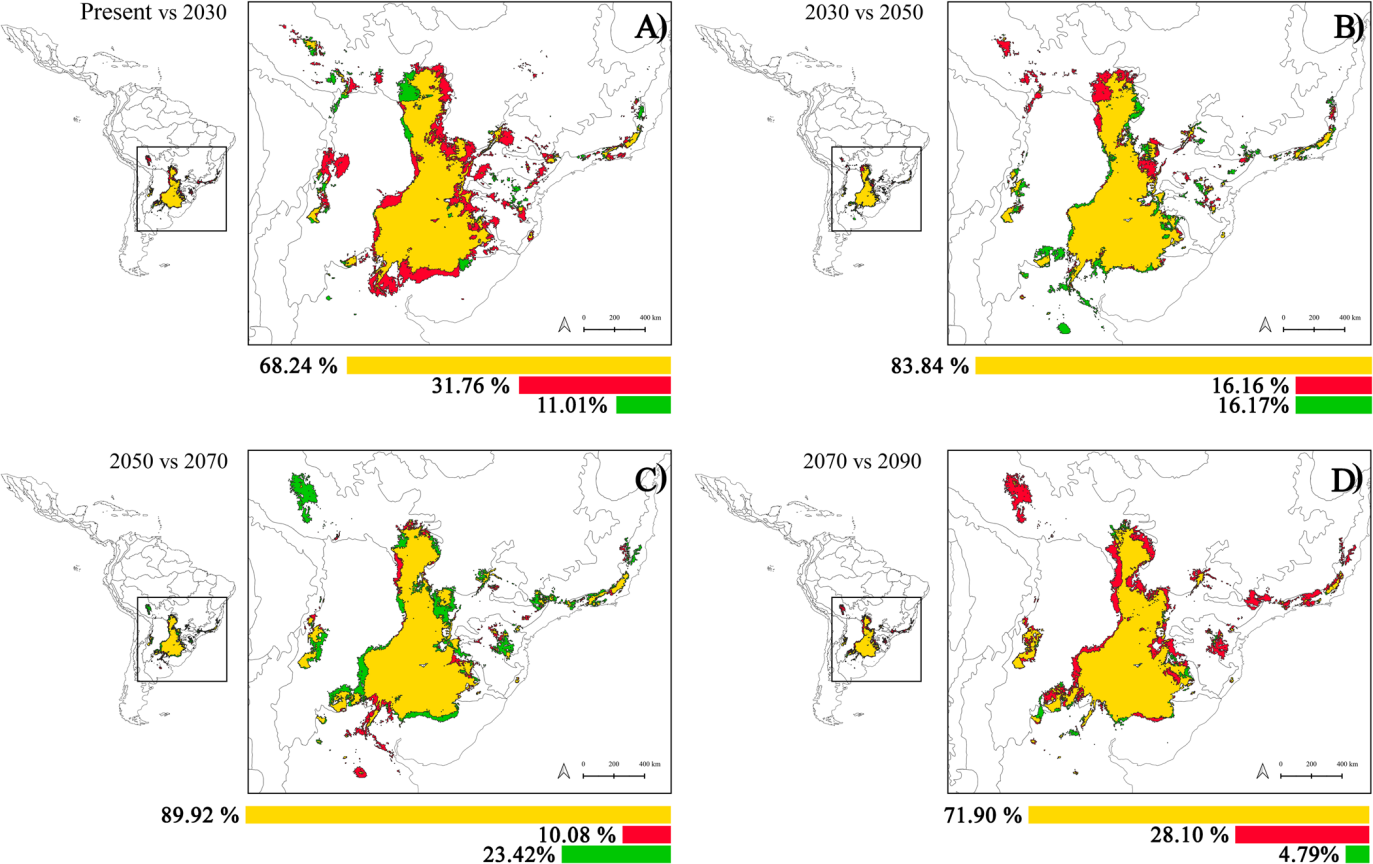
Variations in climatically suitable areas projected for *Alouatta caraya* over successive 20‐year intervals: (A) present to 2030; (B) 2030 to 2050; (C) 2050 to 2070; (D) 2070 to 2090. Bars show the percentage of maintained (yellow), lost (red), and gained (green) areas along each interval.

According to our consensus model, the total suitable area currently available for *A. caraya* encompasses 1,009,213 km^2^, but this value is projected to decrease over time, reaching 686,248 km^2^ by 2090, which represents a 39.43% reduction (Table 2). This decrease occurs gradually across the evaluated timeframe but at heterogeneous rates. During the 2030–2050 interval, our models predict the maintenance of 83.84% of the species' potential distribution area, along with a 16.17% gain in suitable areas (Table 2 and Figure 1). In the next interval (2050–2070), projections suggest that ~90% of the species' distribution area will be retained, along with a substantial gain in the climatically suitable area, reaching 23.42% (Table 2 and Figure 1). Finally, ENMs reconstructed for the 2070–2090 interval predict that ~72% of the species' suitable area will be retained. However, the most concerning scenario occurs when we compare present conditions with projections for the next 20 years (2030), which predict a loss of suitable areas that reaches 31.76% (Figure 1).

**Table 2:** Areas (in km^2^) projected to be maintained, lost, and gained in future scenarios, based on comparisons between the present and each future 20‐year interval (top panel) or between successive 20‐year intervals (middle and bottom panels).

Comparisons	Period 1	Period 2	Area period 1	Area period 2	Area maintained (%)	Area lost (%)	Area gained (%)
Present vs. future projections, without considering dispersal restrictions	Present	2030	1,009,213	773,872	688,672 (68.24)	320,542 (31.76)	85,200 (8.44)
		2050		773,989	689,851(68.36)	319,363 (31.64)	84,137 (8.34)
		2070		908,768	727,331 (72.07)	281,882 (27.93)	181,437 (17.98)
		2090		686,248	611,285 (60.57)	397,928 (39.43)	74,963 (7.43)
Changes in 20‐year timeframes, without considering dispersal restrictions	Present	2030	1,009,213	773,872	688,672 (68.24)	320,542 (31.76)	85,200 (11.01)
	2030	2050	773,872	773,989	648,810 (83.84)	125,063 (16.16)	125,179 (16.17)
	2050	2070	773,989	908,768	695,935 (89.92)	78,050 (10.08)	212,833 (23.42)
	2070	2090	908,768	686,248	653,401 (71.90)	255,366 (28.10)	32,847 (4.79)
Changes in 20‐year timeframes, adjusting for the maximum dispersal distance (MDD)	Present	2030	667,277	617,976	533,913 (80.01)	133,343 (19.98)	43,200 (6.99)
	2030	2050	617,976	600,358	549,906 (88.99)	34,336 (5.56)	50,451 (8.40)
	2050	2070	600,358	715,749	592,921 (98.76)	98,186 (16.35)	122,823 (17.16)
	2070	2090	715,749	584,719	570,950 (79.77)	171,496 (23.96)	13,769 (2.35)

*Note:* In the bottom panel, areas were adjusted by the maximum dispersal distance (MDD) measured for *Alouatta caraya*.

When we subtract the lost area from the gained area, we obtain negative values for almost all periods (except for the 2050–2070 interval), showing a general trend of reduction in the climatically suitable area of *A. caraya* from the present to 2100 (Table 2). In all future scenarios, both in comparisons between the present and the future projections and across each 20‐year interval (Table 2), there is a loss of 10%–39% in the total suitable areas for *A. caraya*, despite several gains in the projected potential distribution, particularly toward the southwest (Figure 1).

**Figure 2:**
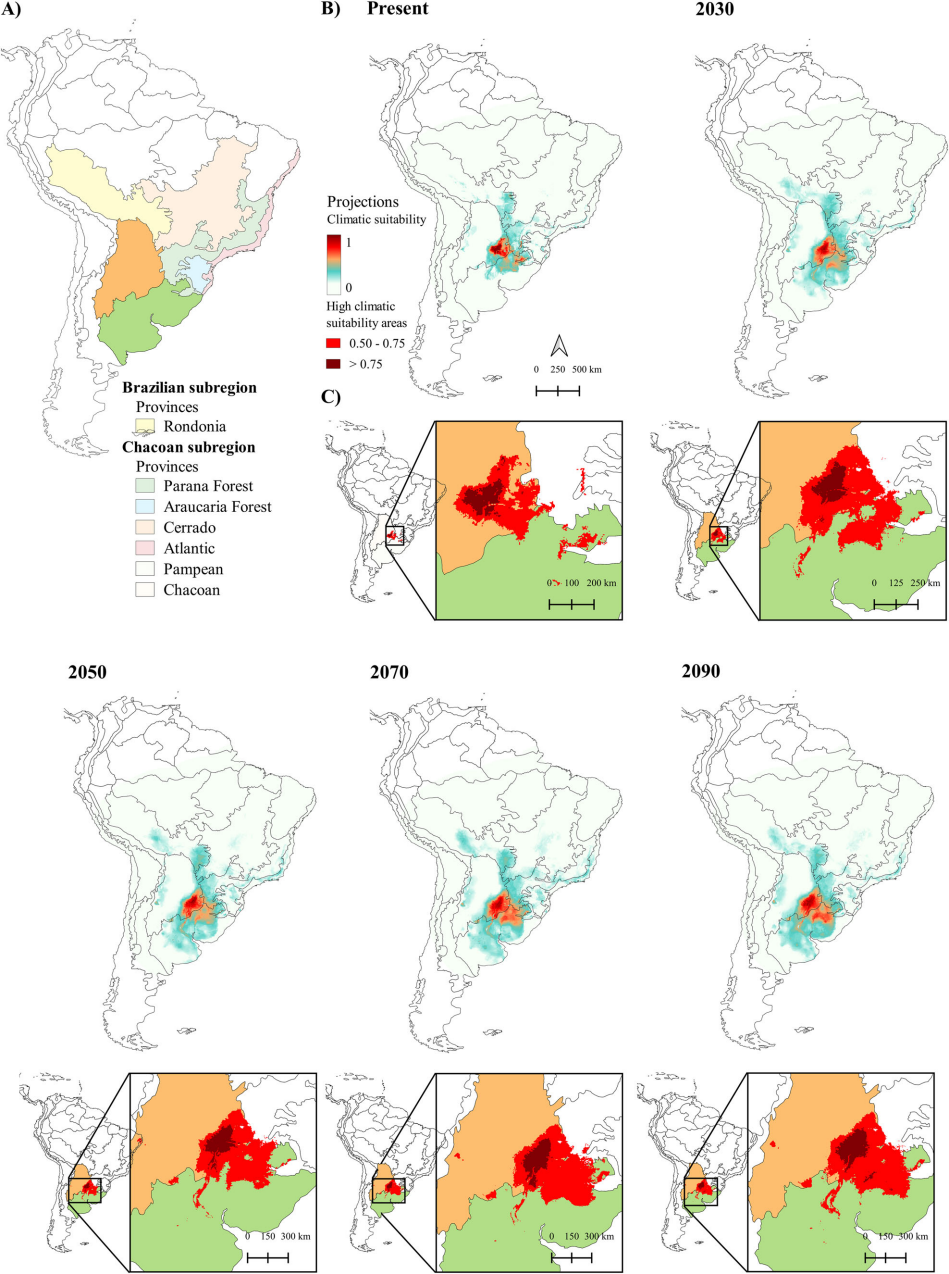
Consensus models showing the distribution of climatically suitable areas across the Neotropics over successive 20‐year intervals. (A) Morrone's biogeographic provinces (Löwenberg‐Neto 2014). (B) Climatically suitable areas projected for different periods. (C) Areas presenting climatic suitability values > 0.5.

Areas with climatic suitability values lower than 0.5 occur in all future scenarios in the Chacoan (Chacoan province, Pampean province, Cerrado province), Parana (Parana Forest province, Araucaria Forest province, Atlantic province), and South Brazilian (Rondônia province) dominions (Figure 2). Two provinces from the Chacoan region exhibit climatic suitability values > 0.5: the Chacoan and Pampean provinces. These regions' suitable areas gradually extend further southeastward from the Chacoan province along the Parana River.

### 3.3 Limits of the Dispersal Extent

**Figure 3:**
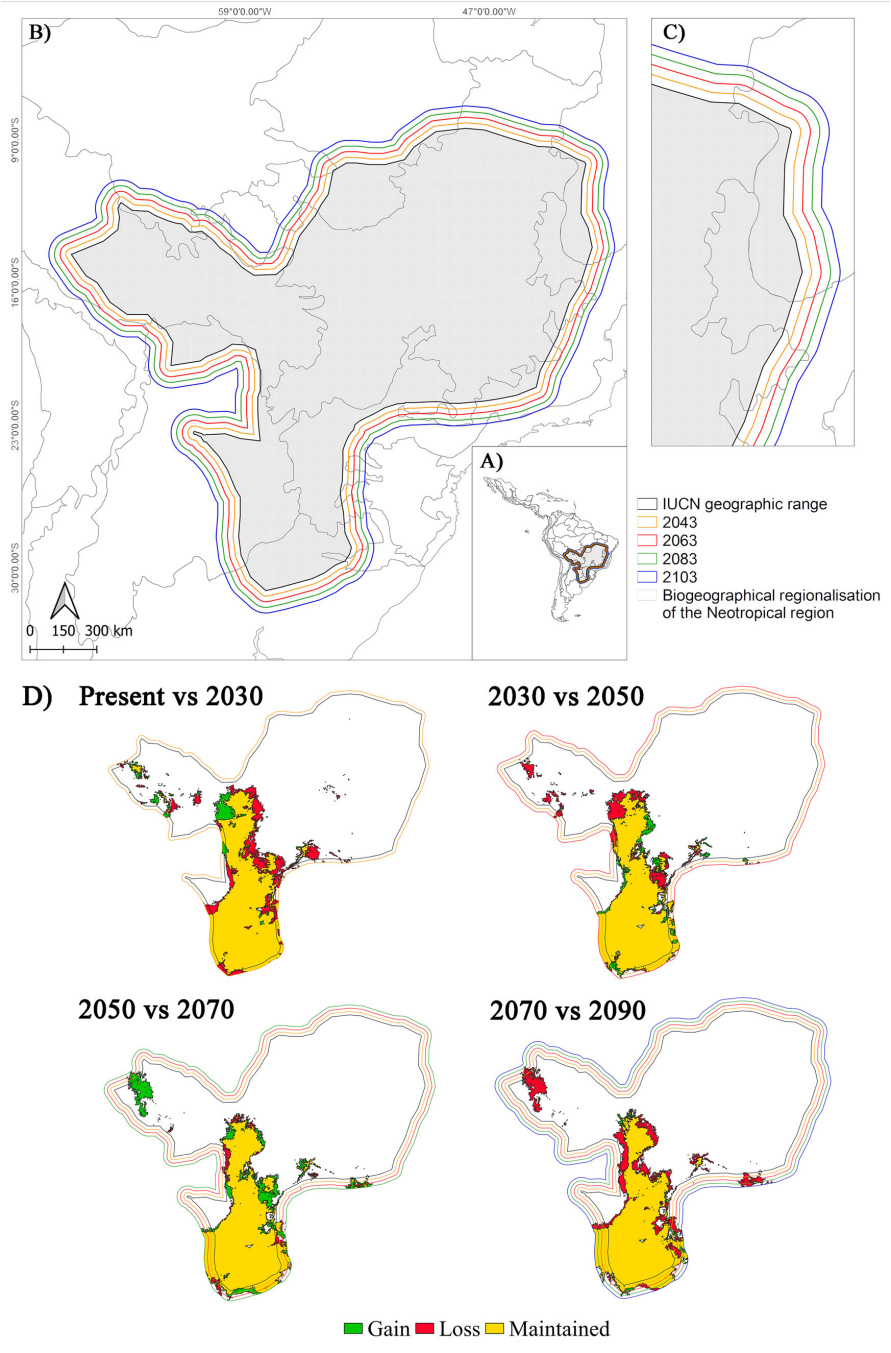
Variations in the climatically suitable area projected for *Alouatta caraya* over successive 20‐year intervals adjusted for the maximum dispersal distance (MDD) of the species. (A) Projection of MDD within the Neotropics. (B and C) Dispersal limits, with an increase of 27,572 m every 20 years. Each color represents a different interval. (D) Climatically suitable areas maintained, lost, and gained across each period, clipped by the MDD of the species.

According to our estimates, the MDD of *A. caraya* over 20‐year intervals (comprising two generations) is 27,572 m (27.57 km). When considering an 80‐year interval, the species can disperse over a maximum distance of 110,288 m (110.29 km) (Figure 3).

Despite the potential gain of suitable areas for the species in future scenarios, these potential ranges drastically decrease when considering dispersal constraints. In fact, using our MDD corrections, we estimated that < 40% of the potentially gained area could be effectively occupied by the species (Table 2).

### 3.4 Conversion, Connectivity, and Fragmentation in the Landscape

Regions with high climatic suitability values have suffered extensive loss of natural vegetation in the last 35 years, with the most significant loss occurring in Brazil (90,118,790 ha), followed by the American Gran Chaco (12,476,732 ha) and the South American Pampa (7,619,502 ha) (Table S2). The conversion of these natural areas can be attributed mainly to pasture management, silviculture, and soybean plantations. Regarding the American Gran Chaco, which, together with the South American Pampa, presents the areas with the highest suitability values (> 0.75) for *A. caraya*, the last 35 years have witnessed a 3.81‐fold increase in the pasture management areas, totaling a conversion of 6,383,280 ha (Table S3). During the same period, there was a 4.38‐fold increase in the area used for silviculture in the Sudamerican Pampa, which presently has reached 2,628,277 ha.

According to our indices (Table 3), both the Gran Chaco and the Sudamerican Pampa have experienced increased levels of fragmentation between 1985 and 2020 (Table 3). Indeed, in both provinces, perimeter measures increased at a faster rate than the total woody area, leading to a decrease in the FI and indicating increased levels of fragmentation. This was especially noteworthy for the Chacoan Province, where the total woody area increased 1.09 times from 1985 to 2020, but perimeter values increased 1.41 times during the same period. Additionally, connectivity between fragments has decreased, as the mean distance between woody fragments gradually increased (Table 3).

**Table 3:** Landscape metrics calculated from 1985 to 2020, in 7‐year intervals, using MapBiomas data.

Provinces	Year	Area (km^2^)	Perimeter (km)	FI	Mean connectivity (km)
Chacoan	1985	245,997	143,608	1.713	474,201
	1992	212,561	136,748	1.554	400,428
	1999	224,587	154,973	1.449	504,066
	2006	233,291	163,328	1.428	507,843
	2013	258,633	188,153	1.375	526,008
	2020	268,704	203,119	1.323	524,436
Pampean	1985	15,149	19,115	0.793	260,650
	1992	16,713	20,664	0.809	267,377
	1999	18,185	22,324	0.815	276,579
	2006	19,827	25,062	0.791	279,221
	2013	20,917	26,350	0.794	284,694
	2020	22,759	29,012	0.784	289,859

*Note:* We measured area and perimeter values considering only woody natural vegetation categories. We estimated the fragmentation index (FI) by dividing the area by the perimeter parameters. We measured mean connectivity as the average distance between the centroids of woody fragments.

**Figure 4:**
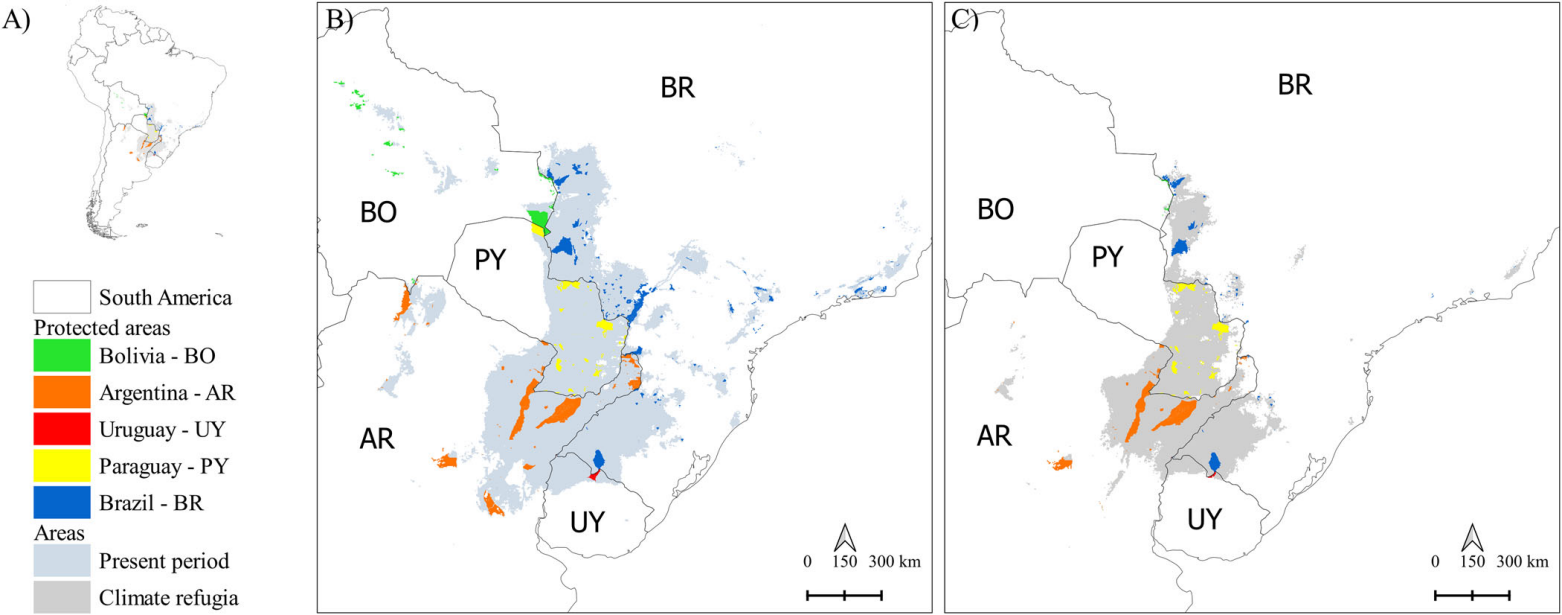
Map of South America (A), indicating the location of protected areas in each country, considering the climatically suitable areas predicted for *A. caraya* in the present (B) or the area that remains climatically suitable for the species over time (climatic refuge) (C).

Protected areas currently comprise ~11% of *A. caraya*'s current potential distribution area, of which 41.5% are found in Argentina and 33.3% in Brazil (Figure 4 and Table S4). In future scenarios, the proportion of currently protected areas overlapping with high‐suitability regions remains between 10.5% and 13.68%. However, the total protected area decreases from 111,159 to 56,751 km^2^ if only climatically stable areas (where populations of the species can be maintained over time) are considered (Figure 4 and Table S4). At present, most of the protected areas that remain climatically suitable for *A. caraya* over time are located in Argentina.

## 4 Discussion

This is the first study to employ different modeling algorithms and a set of future projections to predict the distribution of climatically suitable areas for *A. caraya* in the context of landscape changes and protected areas. Our results suggest that the target species may be under more severe threats than currently considered. The predicted extent of *A. caraya*'s climatically suitable area under current conditions is notably smaller than both the inferred distribution range reported by the IUCN (2023) and the suitable areas projected by Holzmann et al. (2015). Moreover, only 11% of *A. caraya*'s potential distribution area is currently protected, turning it one of the primate species with the lowest levels of protected areas coverage (Agostini et al. 2022; Gomes et al. 2024). Future projections make this scenario even worse, suggesting a continuous decrease in the extent of *A. caraya*'s climatically suitable area from 2021 to 2100. Although a gain in the suitable area was also inferred in some periods, we showed herein that most of this area cannot be reached by the black‐and‐gold howler monkey due to time‐dispersal constraints in the face of the rapid landscape changes.

If we consider the time window proposed by the A3 criterion of the IUCN (IUCN 2023) (population reductions projected, inferred, or suspected to occur within the next three generations), which for black howler monkeys extends until 2050, the cumulative projected habitat loss reaches 31.64%, thereby warranting the Vulnerable category for the species. Thus, we herein endorse the claims of Oklander et al. (2017) that *A. caraya* is already facing serious risks related to habitat loss, fragmentation, and isolation, which justifies a review of its conservation status.

### 4.1 Current Requirements and Distribution

As valuable tools in ecology, biogeography, and conservation (Whittaker et al. 2005; Jetz et al. 2008), ENMs can be employed to identify which bioclimatic layers influence a species' distribution, providing important insights into its climatic requirements (Amiri et al. 2020; Linchamps et al. 2023). We found that isothermality and mean diurnal range are the key variables influencing the distribution of the black‐and‐gold howler monkey. For both variables, the species seems to be better adapted to low to intermediate temperature values, or individuals actively select areas presenting moderate climatic conditions (i.e., avoiding extremes). Our results corroborate those of Holzmann et al. (2015), who identified temperature range and minimum temperatures as key factors for the distributions of *A. caraya* and *A. guariba* across South America. These findings support the potential vulnerability of *A. caraya* to temperature changes, which may not only restrict the species' geographical distribution but also alter foraging and mating behaviors, driving changes in morphological and physiological traits as a form of adaptation (Hetem et al. 2014; Sherwin et al. 2013; Raman et al. 2020). In this context, we cannot precisely predict the physiological effects (and their demographic consequences) if *A. caraya* had to occupy areas with more extreme bioclimatic conditions in the future.

Holzmann et al. (2015) employed MaxEnt to model the potential distribution of *A. caraya* for the period 1970–2000 and estimated an extent of 8,248,716 km^2^ of suitable area for the species. This area is eight times larger than predicted by our study. This disagreement can be attributed to inherent limitations of studies relying on existing data sources, such as differences in the number and distribution of occurrences (van Proosdij et al. 2016), variations in the way pseudo‐absences/absences are obtained or treated (Barbet‐Massin et al. 2012), the use of distinct methodological approaches (Hallgren et al. 2019), and the implementation or not of consensus (ensemble) models (Araújo and New 2007; Meller et al. 2014). It has already been shown that some algorithms, such as MaxEnt, can overpredict species distribution, particularly if default regularization parameters are used (Cao et al. 2013). Thus, applying varied algorithms in our modeling strategy and synthesizing their results through an ensemble helped us overcome several of these limitations (Araújo and New 2007; Meller et al. 2014).

Nevertheless, our study also aligns with Holzmann et al. (2015) in some respects. In both cases, the areas with the highest values of climatic suitability predicted for *A. caraya* occurred southeast of the Chacoan province, along the Parana River. The Chacoan province is characterized by a mosaic of semi‐open habitats, including natural grasslands, non‐native pastures, and silvopastoral systems (Grau et al. 2015; Marinaro et al. 2022), which seem to provide a suitable habitat for *A. caraya* considering their climatic and biotic characteristics. Nevertheless, we have shown herein that this province is undergoing rapid reductions in woody environments, coupled with increased fragmentation and isolation, which are very likely to impose new threats to the species' persistence.

### 4.2 Applications for Conservation

We found that climate change over the next 80 years may impose a reduction of up to 39.43% on the extent of *A. caraya*'s current climatically suitable area. In different timeframes, projections suggest a trend toward higher losses than gains in suitable areas, as has also been predicted for other Neotropical primates (Pinto et al. 2023). This impact may be further exacerbated when considering the combined effects of habitat loss driven by climate change and ongoing forest fragmentation. Indeed, much of the potentially gained areas are distributed in a fragmented way far beyond the MDD of the species. This fragmentation hinders dispersal, potentially altering population dynamics and, consequently, reducing genetic diversity (Oklander et al. 2022). Ultimately, maintaining populations over time requires habitats with sufficient geographic extent and connectivity to support the ecological and functional dynamics of the environment (Arroyo‐Rodríguez and Mandujano 2009).

Since in situ conservation strategies may be crucial to mitigate the effects of climate change as well as the conversion of natural habitats (Cabral Rezende et al. 2020; Gonçalves‐Souza et al. 2021), expanding the protected area network by establishing reserves in strategic locations for *A. caraya* is imperative. Our results show that the extent of protected areas within the species' suitable ranges—11% for the present and ~10.5%–13.6% for the future—is not sufficient to counteract habitat loss. This concern becomes even greater when we consider only climatically suitable areas that are maintained throughout the evaluated timeframe. In fact, these climatic refuges encompass just half of the protected areas currently available (Table S4). As connectivity between protected areas is likely to decrease, we expect a decline in their effectiveness for species conservation.

As a result, we suggest that mitigation strategies for the species' threats should focus on two key actions: first, expand protection in existing conservation areas, especially in regions with greater habitat suitability and stability; then, create new protected areas and ecological corridors to maintain habitat connectivity. Genetic studies have already demonstrated high levels of population structure in *A. caraya* (Ascunce et al. 2007; Oklander et al. 2017; 2022), reinforcing the idea that each population holds intrinsic value. In this scenario, losing any individual population due to climate change or habitat conversion could cause irretrievable damage to the species' genetic pool. Nevertheless, given spatial, temporal, and financial constraints, prioritizing climatically stable areas becomes crucial not only for the long‐term persistence of populations but also for preserving higher levels of genetic diversity (Carnaval et al. 2009), which may be key for populations to adapt to the new environmental conditions driven by ongoing habitat changes. Based on our findings, we recommend creating and expanding conservation units within the Gran Chaco and South American Pampa provinces, which harbor the most climatically suitable and stable areas for *A. caraya*. However, to ensure the long‐term persistence of multiple *A. caraya* populations, it is also essential to preserve connectivity within these provinces, reinforcing the need for ecological corridors that facilitate gene flow and promote dispersal between fragments, buffering the negative effects of fragmentation.

Future studies should address current gaps and limitations of this study, particularly the use of HR estimates as a proxy for dispersal capacity, which may overestimate movement potential in highly fragmented landscapes. Although genetic data for *A. caraya* remains limited due to sampling constraints, using phylogeographic approaches will be essential to improve our understanding of dispersal dynamics and population connectivity. Even so, by combining satellite‐derived images, scientifically informed platforms, and different modeling algorithms under various climate scenarios, our study provides robust evidence supporting a trend toward habitat loss (both predicted and projected) for *A. caraya*. Our findings concerning the impact of climate change, together with the increasing levels of fragmentation and isolation, underscore the need to revisit the species' current “Least Concern” conservation status (IUCN 2023). Finally, beyond serving as a proxy for population decline under IUCN Subcriterion C (IUCN 2022), ENMs also provide valuable insights for guiding conservation planning.

## Author Contributions

**Jéssyca B. Schwantes:** conceptualization (lead), data curation (equal), formal analysis (equal), methodology (equal), writing – original draft (equal). **Lucas A. Antunes:** formal analysis (equal), methodology (equal), writing – original draft (equal). **Vanessa B. Fortes:** supervision (equal), validation (equal), writing – review and editing (supporting). **Lizandra J. Robe:** formal analysis (equal), methodology (supporting), supervision (lead), validation (equal), writing – review and editing (lead). All the authors commented on and approved the final version of the manuscript.

## Conflicts of Interest

The authors declare no conflicts of interest.

## Supporting information


**Figure S1:** Consensus models showing the distribution of *A. caraya*'s climatically suitable areas, as projected for different future 20‐year intervals considering three Shared Socio‐economic Pathways (SSP): 2‐4.5 (optimistic), 3‐7.0 (intermediate), and 5‐8.5 (pessimistic). The threshold values of 0.33, 0.66, and 0.99 represent the suitable areas recovered under one, two, and three of the different SSP projection models, respectively.
**Figure S2:** A) Relative contribution of each of the seven bioclimatic variables employed in the modeling strategy. B) Species response curves for each bioclimatic variable. The X‐axis represents the environmental gradient, whereas the Y‐axis indicates the suitability scores predicted by the ENMs.
**Table S1:** Areas (in km^2^ and %) projected to be maintained, lost, or gained under different future scenarios, based on comparisons between the present and each future 20‐year interval. In each case, future projections were reconstructed using BCC and CMCC Global Climate Models (GCMs) under three Shared Socio‐economic Pathways (SSPs).
**Table S2:** Extent of natural vegetation estimated for the American Gran Chaco, the Sudamerican Pampa, and Brazil at 7‐year intervals from 1985 to 2020, based on MapBiomas data.
**Table S3:** Extent of area converted to grasslands in the Chaco and silviculture in the Pampa, as estimated from MapBiomas data at 7‐year intervals from 1985 to 2020.
**Table S4:** Extent of protected area within *A. caraya* potential distribution range, as projected for the present, each future 20‐year interval, and identified climate refuges, subdivided by country.

## Data Availability

The data used to perform the ecological niche modeling analyses can be requested from the authors. We have data from restricted platforms such as SALVE (IBAMA/Brazil) because we include species threatened by illegal trafficking or hunting.
