# Supplementary material for: Present and Future: Using Ecological Niche Modeling to Understand the Conservation Status of Alouatta caraya (Primates, Atelidae) and Promote Its Protection
Source: Am J Primatol. 2025 Aug 19;87(8):e70066. doi: 10.1002/ajp.70066 (PMC12364432; doi:10.1002/ajp.70066)
Supplement: Supplementary file 1 — Figure S1: Consensus models showing the distribution of A. caraya's climatically suitable areas, as projected for different future 20‐year intervals considering three Shared Socio‐economic Pathways (SSP): 2‐4.5 (optimistic), 3‐7.0 (intermediate), and 5‐8.5 (pessimistic). The threshold values of 0.33, 0.66, and 0.99 represent the suitable areas recovered under one, two, and three of the different SSP projection models, respectively. Figure S2: A) Relative contribution of each of the seven bioclimatic variables employed in the modeling strategy. B) Species response curves for each bioclimatic variable. The X‐axis represents the environmental gradient, whereas the Y‐axis indicates the suitability scores predicted by the ENMs. Table S1: Areas (in km2 and %) projected to be maintained, lost, or gained under different future scenarios, based on comparisons between the present and each future 20‐year interval. In each case, future projections were reconstructed using BCC and CMCC Global Climate Models (GCMs) under three Shared Socio‐economic Pathways (SSPs). Table S2: Extent of natural vegetation estimated for the American Gran Chaco, the Sudamerican Pampa, and Brazil at 7‐year intervals from 1985 to 2020, based on MapBiomas data. Table S3: Extent of area converted to grasslands in the Chaco and silviculture in the Pampa, as estimated from MapBiomas data at 7‐year intervals from 1985 to 2020. Table S4: Extent of protected area within A. caraya potential distribution range, as projected for the present, each future 20‐year interval, and identified climate refuges, subdivided by country. [file AJP-87-e70066-s001.docx]

**Supplementary material**

Present and future: Using ecological niche modeling to understand the conservation status of Alouatta caraya (Primates, Atelidae) and promote its protection

Schwantes, J. B., Antunes, L. A., Fortes, V. B., Robe, L. J.

Table S1. Areas (in km^2^ and %) projected to be maintained, lost, or gained under different future scenarios, based on comparisons between the present and each future 20-year interval. In each case, future projections were reconstructed using BCC and CMCC Global Climate Models (GCMs) under three Shared Socio-economic Pathways (SSPs).

| GCM | Period | SSP | Total area | Maintained area | % | Area lost | % | Area gained | % |
| --- | --- | --- | --- | --- | --- | --- | --- | --- | --- |
|  | Current |  | 1,009,213 |  |  |  |  |  |  |
| BCC | 2030 | 245 | 1,010,571 | 692,763 | 68.64 | 316,450 | 31.36 | 317,807 | 31.49 |
|  | 2030 | 370 | 1,752,416 | 893,805 | 88.56 | 115,409 | 11.44 | 858,611 | 85.08 |
|  | 2030 | 585 | 1,411,807 | 753,311 | 74.64 | 255,903 | 25.36 | 658,496 | 65.25 |
| CMCC | 2030 | 245 | 970,297 | 705,307 | 69.89 | 303,906 | 30.11 | 264,980 | 26.26 |
|  | 2030 | 370 | 1,195,374 | 814,095 | 80.67 | 195,119 | 19.33 | 381,281 | 37.78 |
|  | 2030 | 585 | 813,725 | 667,740 | 66.16 | 341,473 | 33.84 | 145,981 | 14.46 |
| BCC | 2050 | 245 | 1,720,625 | 863,292 | 85.54 | 145,922 | 14.46 | 857,333 | 84.95 |
|  | 2050 | 370 | 1,556,205 | 779,371 | 77.23 | 229,862 | 22.78 | 776,835 | 76.97 |
|  | 2050 | 585 | 1,827,051 | 864,262 | 85.64 | 144,952 | 14.36 | 962,789 | 95.40 |
| CMCC | 2050 | 245 | 1,275,782 | 815,274 | 80.78 | 193,940 | 19.22 | 460,507 | 45.63 |
|  | 2050 | 370 | 1,096,315 | 767,794 | 76.08 | 241,420 | 23.92 | 328,520 | 32.55 |
|  | 2050 | 585 | 1,089,529 | 749,260 | 74.24 | 259,955 | 25.76 | 340,284 | 33.72 |
| BCC | 2070 | 245 | 2,077,215 | 929,627 | 92.11 | 79,587 | 7.89 | 1,147,587 | 113.71 |
|  | 2070 | 370 | 1,856,652 | 856,847 | 84.90 | 152,367 | 15.10 | 999,804 | 99.07 |
|  | 2070 | 585 | 2,335,109 | 918,364 | 91.00 | 90,850 | 9.00 | 1,416,745 | 140.38 |
| CMCC | 2070 | 245 | 1,122,729 | 775,014 | 76.79 | 234,200 | 23.21 | 347,715 | 34.45 |
|  | 2070 | 370 | 1,106,680 | 756,314 | 74.94 | 252,899 | 25.06 | 350,366 | 34.72 |
|  | 2070 | 585 | 1,061,862 | 718,452 | 71.19 | 290,762 | 28.81 | 343,410 | 34.03 |
| BCC | 2090 | 245 | 1,949,963 | 912,088 | 90.38 | 97,127 | 9.62 | 1,037,875 | 102.84 |
|  | 2090 | 370 | 1,885,218 | 870,965 | 86.30 | 138,249 | 13.70 | 1,014,253 | 100.50 |
|  | 2090 | 585 | 2,253,776 | 902,196 | 89.40 | 107,018 | 10.60 | 1,351,580 | 133.92 |
| CMCC | 2090 | 245 | 1,221,478 | 777,575 | 77.05 | 231,639 | 22.95 | 443,903 | 43.99 |
|  | 2090 | 370 | 880,487 | 652,667 | 64.67 | 356,547 | 35.33 | 227,820 | 22.57 |
|  | 2090 | 585 | 525,864 | 369,825 | 36.64 | 639,389 | 63.36 | 156,039 | 15.46 |

Table S2. Extent of natural vegetation estimated for the American Gran Chaco, the Sudamerican Pampa, and Brazil at 7-year intervals from 1985 to 2020, based on MapBiomas data.

| Years | Chaco (Ha) | % | Pampa (Ha) | % | Brazil (Ha) | % |
| --- | --- | --- | --- | --- | --- | --- |
| 1985 | 99,008,870 | 92 | 63,207,097 | 58 | 658,388,119 | 77 |
| 1992 | 97,210,524 | 90 | 63,241,464 | 58 | 636,848, 210 | 75 |
| 1999 | 95,299,625 | 88 | 62,180,963 | 57 | 613,859,846 | 72 |
| 2006 | 92,154,617 | 85 | 58,199,700 | 53 | 590,403,588 | 69 |
| 2013 | 89,040,734 | 82 | 55,962,349 | 51 | 579,931,895 | 68 |
| 2020 | 86,532,138 | 80 | 55,587,595 | 51 | 568,269,329 | 66 |
| 1985-2020 | -12,476,732 |  | -7,619,502 |  | -90,118,790 |  |

Table S3. Extent of area converted to grasslands in the Chaco and silviculture in the Pampa, as estimated from MapBiomas data at 7-year intervals from 1985 to 2020.

|  | Conversion Area | | | |
| --- | --- | --- | --- | --- |
| Province | Most common type of conversion | Year | Area (Ha) | Difference in 35 years |
| Chaco | Grasslands areas | 1985 | 2,272,128 | 6,383,280 ha |
|  |  | 1992 | 3,411,329 |  |
|  |  | 1999 | 3,983,993 |  |
|  |  | 2006 | 5,124,672 |  |
|  |  | 2013 | 6,530,399 |  |
|  |  | 2020 | 8,655,408 |  |
| Pampa | Silviculture area | 1985 | 600,232 | 2,028,045 ha |
|  |  | 1992 | 794,175 |  |
|  |  | 1999 | 986,049 |  |
|  |  | 2006 | 1,540,095 |  |
|  |  | 2013 | 2,248,639 |  |
|  |  | 2020 | 2,628,277 |  |

Table S4. Extent of protected area within *A. caraya* potential distribution range, as projected for the present, each future 20-year interval, and identified climate refuges, subdivided by country.

| Period | Total area (km²) | Country | Protected area (km^2^) | %1 | %2 |
| --- | --- | --- | --- | --- | --- |
| **Current** | 1,009,213 | Argentina | 46,176 | 4.58 | 41.5 |
|  |  | Bolivia | 12,508 | 1.24 | 11.3 |
|  |  | Brazil | 37,069 | 3.67 | 33.3 |
|  |  | Paraguay | 14,285 | 1.42 | 12.9 |
|  |  | Uruguay | 1,121 | 0.11 | 1 |
|  |  | **Total** | **111,159** | **11.01** |  |
| **2030** | 773,872 | Argentina | 37,544 | 4.85 | 35.47 |
|  |  | Bolivia | 26,775 | 3.46 | 25.3 |
|  |  | Brazil | 26,919 | 3.48 | 25.44 |
|  |  | Paraguay | 14,175 | 1.83 | 13.39 |
|  |  | Uruguay | 420 | 0.05 | 0.4 |
|  |  | **Total** | **105,833** | **13.68** |  |
| **2050** | 773,989 | Argentina | 43,476 | 8.26 | 73.1 |
|  |  | Bolivia | 6,868 | 1.31 | 11.55 |
|  |  | Brazil | 24,415 | 4.64 | 41.05 |
|  |  | Paraguay | 12,223 | 2.32 | 20.55 |
|  |  | Uruguay | 497 | 0.09 | 0.84 |
|  |  | **Total** | **87,480** | **11.3** |  |
| **2070** | 908,768 | Argentina | 40,623 | 4.47 | 42.56 |
|  |  | Bolivia | 13,630 | 1.5 | 14.28 |
|  |  | Brazil | 28,765 | 3.17 | 30.14 |
|  |  | Paraguay | 11,328 | 1.25 | 11.87 |
|  |  | Uruguay | 1,094 | 0.12 | 1.15 |
|  |  | **Total** | **95,441** | **10.5** |  |
| **2090** | 686,248 | Argentina | 37,782 | 5.51 | 50.63 |
|  |  | Bolivia | 6,831 | 1 | 9.15 |
|  |  | Brazil | 18,070 | 2.63 | 24.21 |
|  |  | Paraguay | 11,058 | 1.61 | 14.82 |
|  |  | Uruguay | 889 | 0.13 | 1.19 |
|  |  | **Total** | **74,629** | **10.87** |  |
| **Climate refugia** | 529,100 | Argentina | 30,056 | 5.68 | 53 |
|  |  | Bolivia | 697 | 0.13 | 1.2 |
|  |  | Brazil | 14,655 | 2.77 | 25.8 |
|  |  | Paraguay | 10,945 | 2.07 | 19.3 |
|  |  | Uruguay | 398 | 0.08 | 0.7 |
|  |  | **Total** | **56,751** | **10.73** |  |

%1 - Percentage based on total area

%2 - Percentage of each country based on percentage 1

Figure S1. Consensus models showing the distribution of *A. caraya*’s climatically suitable areas, as projected for different future 20-year intervals considering three Shared Socio-economic Pathways (SSP): 2-4.5 (optimistic), 3-7.0 (intermediate), and 5-8.5 (pessimistic). The threshold values of 0.33, 0.66, and 0.99 represent the suitable areas recovered under one, two, and three of the different SSP projection models, respectively. ​​​​


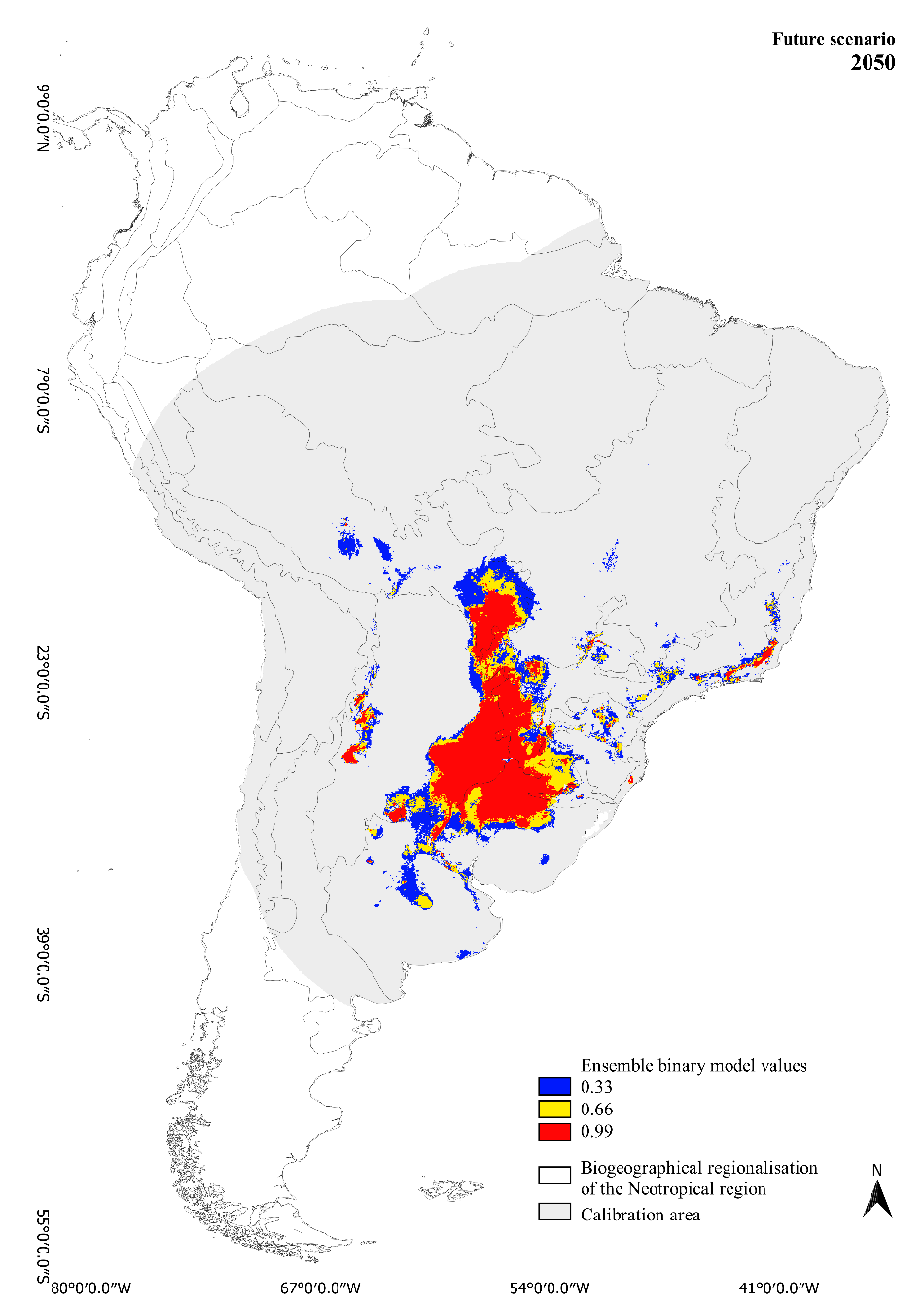

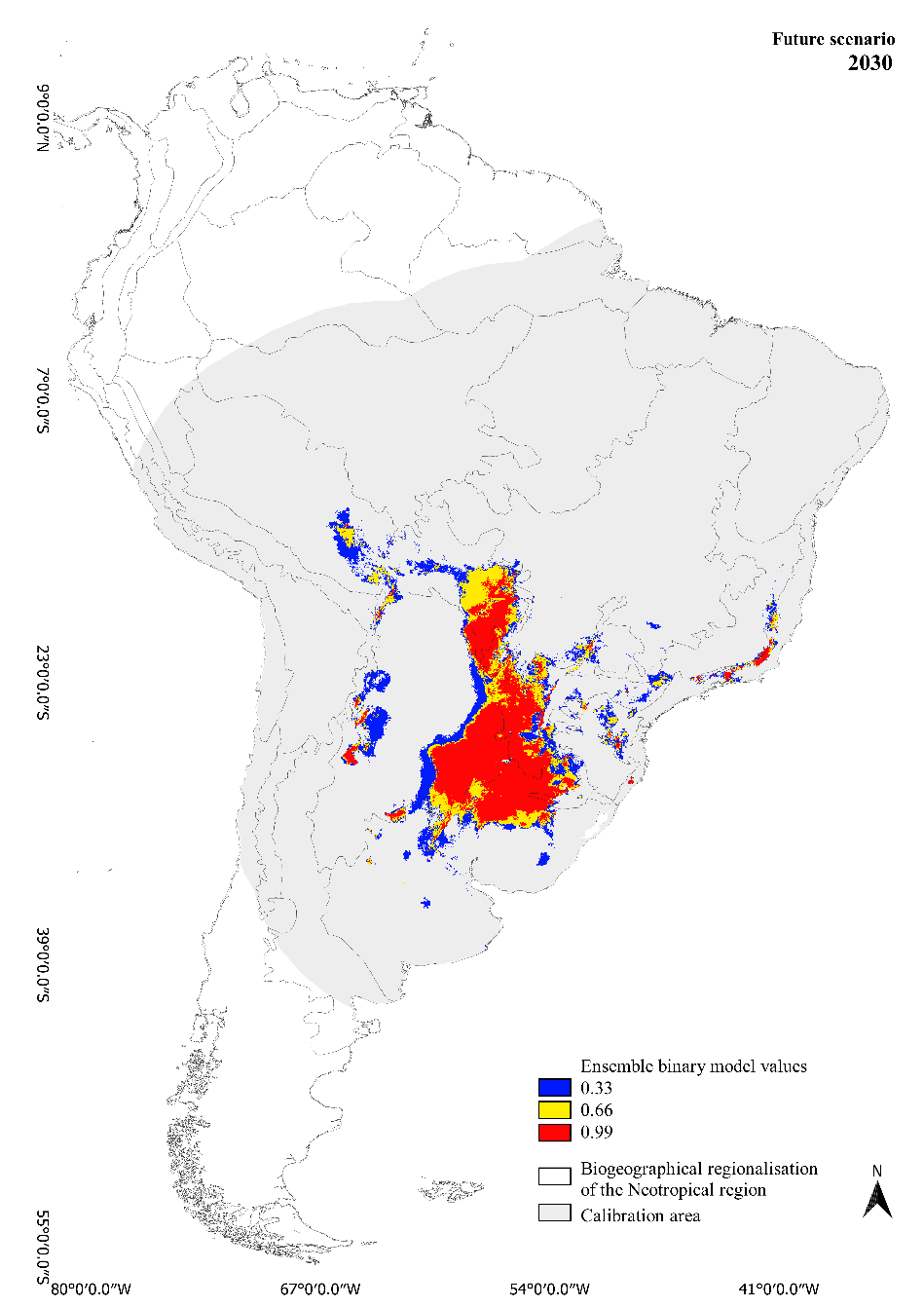


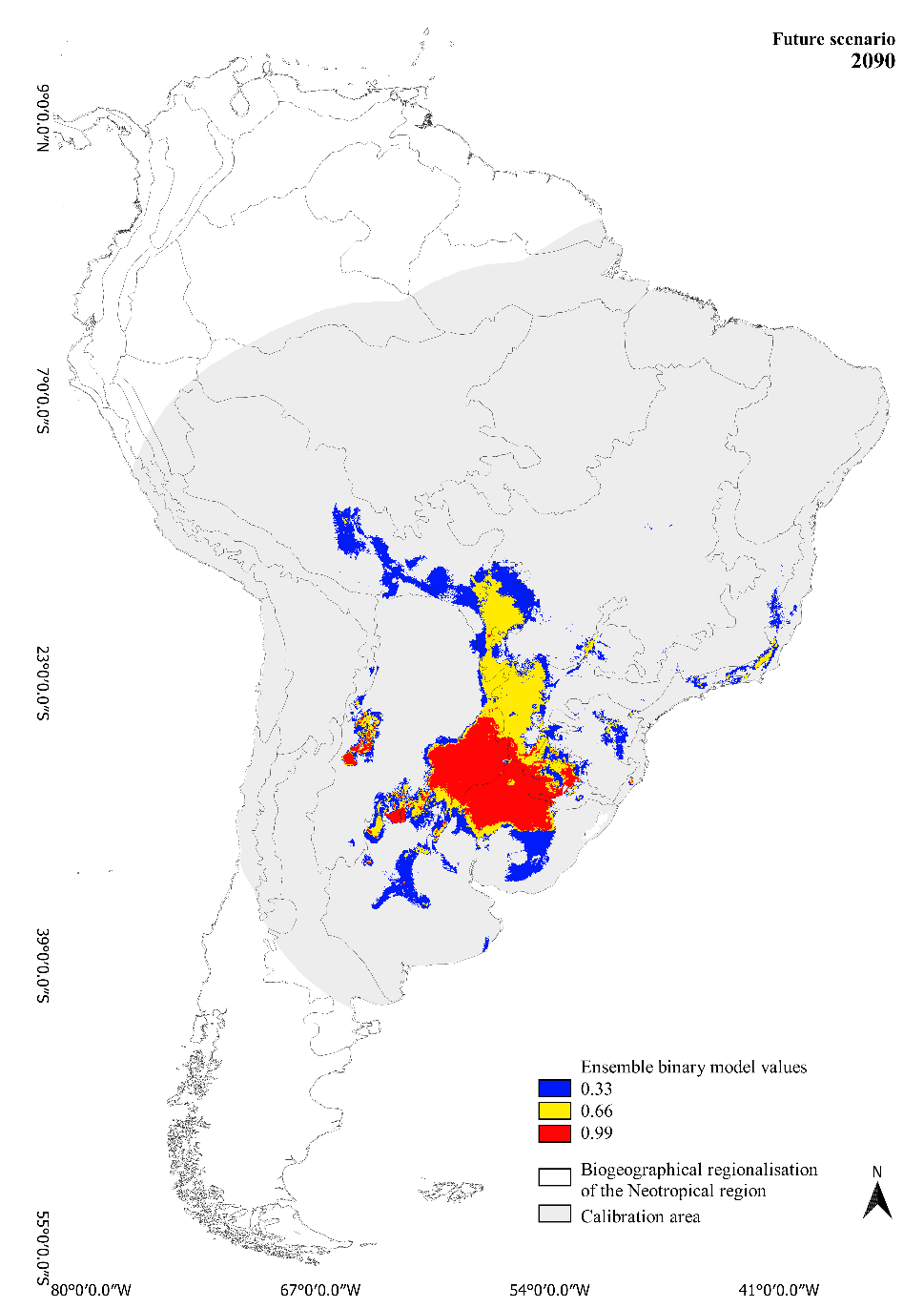

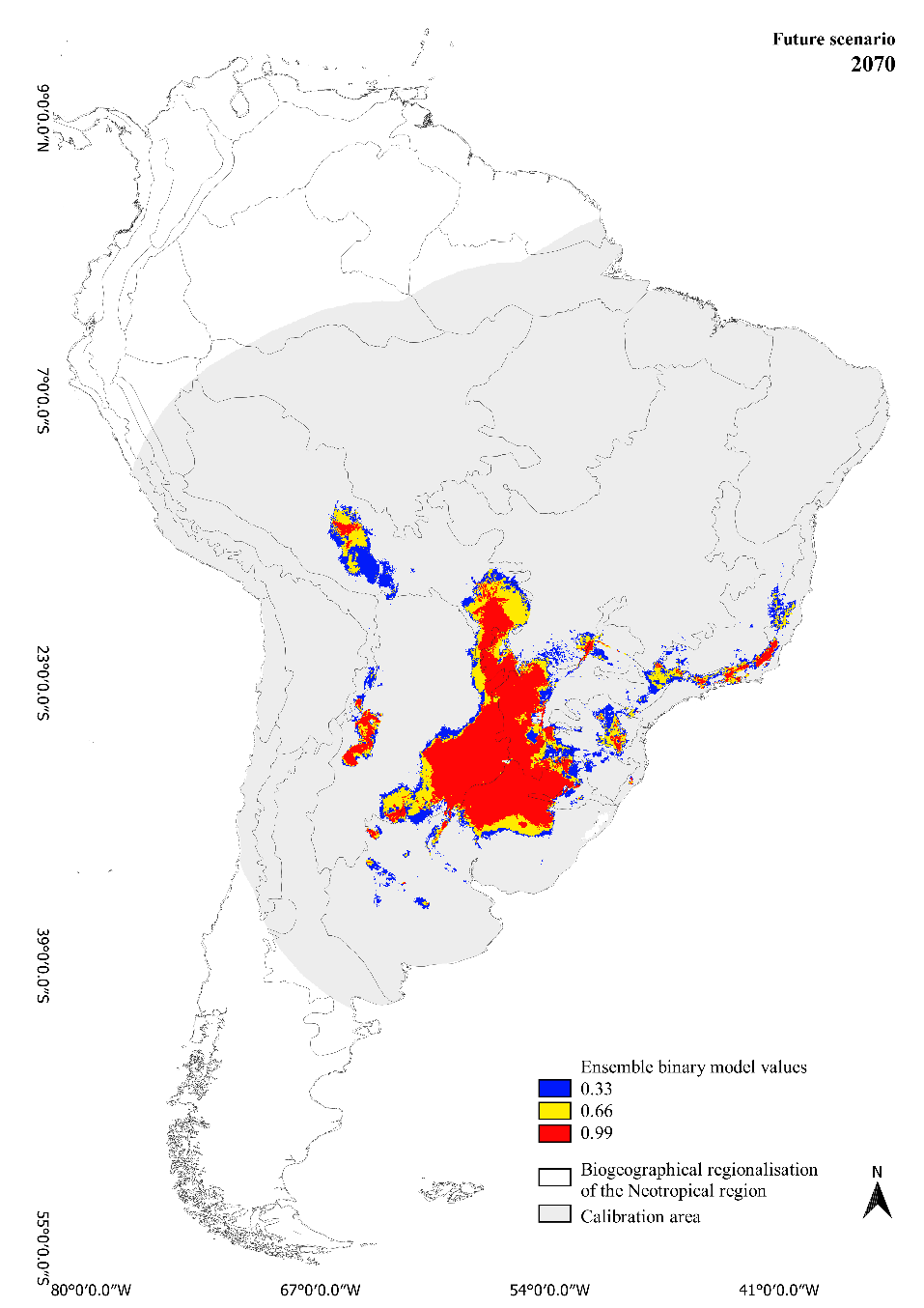


Figure S2. A) Relative contribution of each of the seven bioclimatic variables employed in the modeling strategy. B) Species response curves for each bioclimatic variable. The X-axis represents the environmental gradient, whereas the Y-axis indicates the suitability scores predicted by the ENMs.


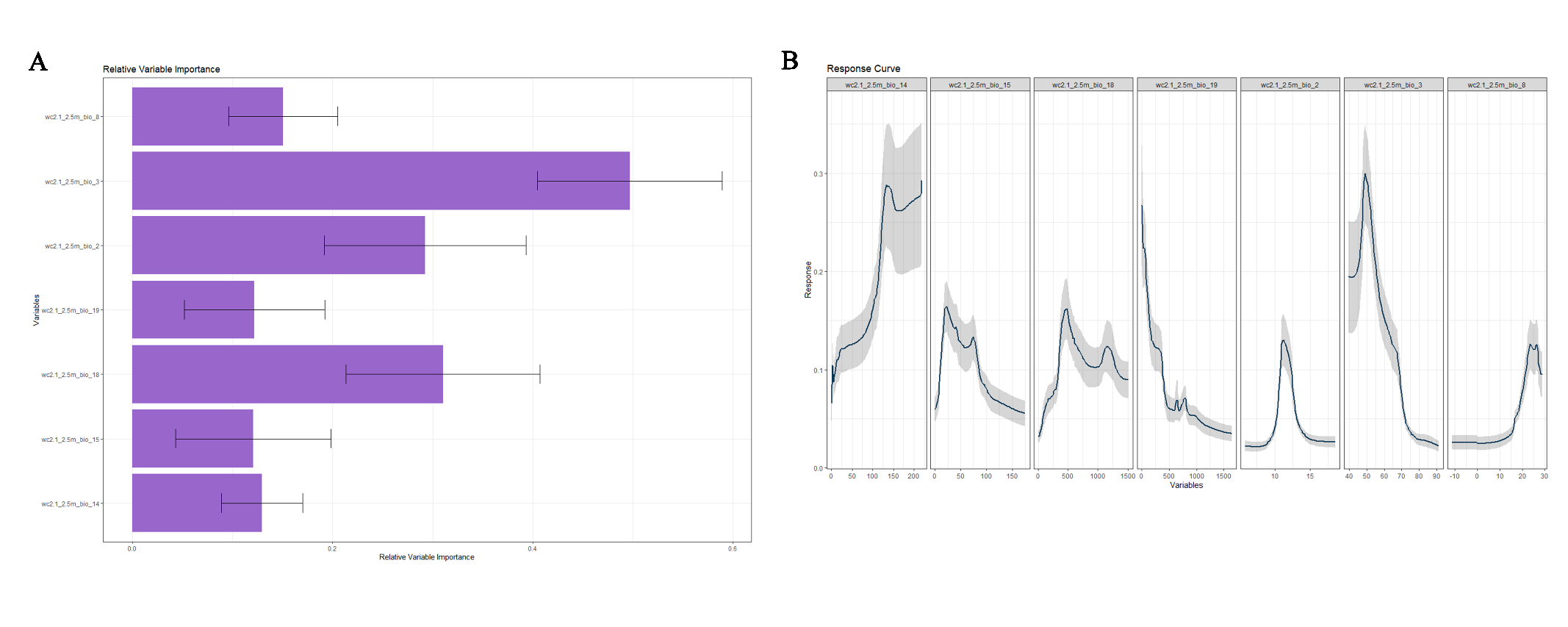
​​​​
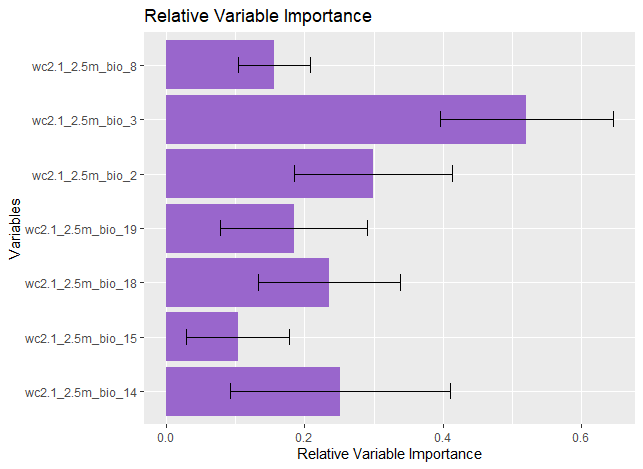


**A**
